# The importance of maintaining the essence of relational medicine in a technological age

**DOI:** 10.1002/jgf2.369

**Published:** 2020-11-28

**Authors:** Sally Lewis

**Affiliations:** ^1^ National Clinical lead for Value‐based healthcare Welsh Government Department for Health Social Services and Children Crown Building Cathays Park Cardiff Cardiff UK; ^2^ Swansea University School of Medicine Swansea Wales UK

I entered general practice in 1998 having originally intended to become a hospital physician. I had felt constrained by the medical hierarchy and the hospital environment and was attracted by the wild frontiers of community medicine, along with the idea of longitudinal care. I qualified in 2000 and immediately became a partner in my training practice, before moving back to a South Wales valley practice in late 2005. I became a GP trainer, a senior partner and was a big advocate of GP independence. Since 2011, I have held other roles too, but that is not important here.

Over the 20 years of my life as a GP, there have been many changes, some good and some not so good. Of those that are not so good, the biggest threat to the philosophy and practice of British generalism are the changes which are pushing us away from relational medicine and toward a more transactional set of interactions with our patients. “The QOF” (the Quality and Outcomes Framework in the new GMS contract 2003/4) was the first major change. It was designed to support the embedding of evidence‐based medicine in primary care and reduce unwarranted variation in practice so that we could improve population outcomes and has since been evaluated, with mixed and hotly debated results.

One of the unintended consequences of QOF was that it started to create a formulaic and rather mechanistic approach to the care of people with chronic disease, drawing us into a trap of “tick box” medicine where the sum of the individual interventions described in the framework were not always greater than the whole. For many patients, we failed to meet their goals and even stopped asking about them in the name of following the evidence‐based formula. This coincided with a proliferation of single disease guidelines which started to become tramlines toward an inevitable destination rather than tools to help us navigate the evidence with our patients and achieve their goals. Many people are living with more than one condition and in a world of increasing subspecialist expertise, patients need generalists to help view the big picture and integrate all information into better decision making and achieving outcomes that matter. Generalism is about coordination, advocacy, and perspective in care. It does not seek to replace subspecialist expertise but is entirely necessary for its success. The basis of generalism in primary care is relational medicine and this is our specialism, along with managing undifferentiated illness and a good deal of uncertainty. Given the evolving complex needs of our population, GPs need to work closely with our generalist cousins in acute medicine and geriatrics if we are to achieve anything that looks at all like integrated medical care for our patients, drawing of course on the expertise from the specialists.

QOF also generated a lot of routine and follow‐up activity in general practice. Intervention always begets more intervention. This, along with increasing complexity and need, and GP numbers falling relative to need, and funding not keeping pace, brought ever more tension between access to appointments and continuity of care.

One response to the access crisis has been to triage all calls into primary care on the phone or online. During the COVID‐19 pandemic, we have seen remote consulting take over completely, sometimes with the addition of video as a helpful adjunct. Have we overegged this? I think so. Many consultations are quite straightforward transactions and can easily and properly be conducted over the phone and with greater convenience for patients. However, some will be disadvantaged by being forced into that approach and that will be hard to measure in a satisfaction score. I also can't help but reflect on those people who have arrived at my surgery and left with a quite unexpected (and sometimes life threatening) diagnosis which undoubtedly would have been missed on the phone. Phone, video, and face‐to‐face consultations are only tools and should be used in a balanced way and none to excess.

By putting up technological barriers, we risk further eroding the relational aspects of our work and the unmeasurable, intangible depth of a face‐to‐face human interaction. I like to go to the waiting room and call people in. You can read so much from the way a person walks across the room. In an age where technology can add so much to our lives, improving outcomes and the sustainability of healthcare systems, let's not make ourselves too inaccessible as human beings. Let’s keep the unmeasurable and intangible but essential relational aspects of our work that enrich our lives and make us better doctors.

## CONFLICT OF INTEREST

The authors have stated explicitly that there are no conflicts of interest in connection with this article.

